# Effectiveness of South Africa's network of protected areas: Unassessed vascular plants predicted to be threatened using deep neural networks are all located in protected areas

**DOI:** 10.1002/ece3.70229

**Published:** 2024-09-02

**Authors:** Bahati Samuel Kandolo, Kowiyou Yessoufou, Mahlatse Kganyago

**Affiliations:** ^1^ Department of Geography, Environmental Management and Energy Studies University of Johannesburg Johannesburg South Africa

**Keywords:** extinction risk assessment, neural networks, plant diversity, protected areas

## Abstract

Globally, we are in the midst of a biodiversity crisis and megadiverse countries become key targets for conservation. South Africa, the only country in the world hosting three biodiversity hotspots within its borders, harbours a tremendous diversity of at‐risk species deserving to be protected. However, the lengthy risk assessment process and the lack of required data to complete assessments is a serious limitation to conservation since several species may slide into extinction while awaiting risk assessment. Here, we employed a deep neural network model integrating species climatic and geographic features to predict the conservation status of 116 unassessed plant species. Our analysis involved in total of 1072 plant species and 96,938 occurrence points. The best‐performing model exhibits high accuracy, reaching up to 83.6% at the binary classification and 56.8% at the detailed classification. Our best‐performing model at the binary classification predicts that 32% (25 species) and 8% (3 species) of Data Deficient and Not‐Evaluated species respectively, are likely threatened, amounting to a proportion of 24.1% of unassessed species facing a risk of extinction. Interestingly, all unassessed species predicted to be threatened are in protected areas, revealing the effectiveness of South Africa's network of protected areas in conservation, although these likely threatened species are more abundant outside protected areas. Considering the limitation in assessing only species with available data, there remains a possibility of a higher proportion of unassessed species being imperilled.

## Graphical abstract

Biodiversity is lost at an unprecedented rate. We need information to inform conservation decisions. All unassessed plants expected to be threatened are located in protected areas in South Africa.

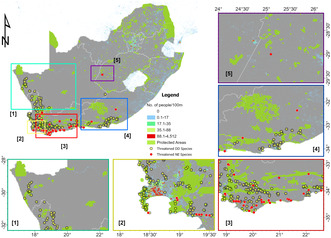

## 1 INTRODUCTION

The conservation of biodiversity is a complex task because of political, scientific and social challenges (Zizka et al., 2021). Unique geographic areas, for example, biodiversity hotspots (Forest et al., 2007) and at‐risk species (Brooks et al., 2004), have to be identified as targets for conservation while considering monetary costs (Gordon et al., 2020; Stuart et al., 2010), community acceptance and time (Balding & Williams, 2016).

To aid in the decision‐making process towards conservation, the International Union for Conservation of Nature (IUCN) red list of threatened species constitutes an indispensable instrument for both researchers and policymakers. It constitutes the most consistent and comprehensive listing of conservation status for plant and animal species in the world (Vié et al., 2009). Nevertheless, plants are not as well represented on the red list as animals, despite their essential ecological role (Stuart et al., 2010) and are sometimes ignored such that charismatic vertebrates are favoured (Balding & Williams, 2016).

The red list categorizes evaluated species according to their geographical range, population trends and unique threats (IUCN, 2019). The categories ‘Least Concern’ (LC) and ‘Near Threatened’ (NT) are considered to be facing no immediate threat, whereas species classified as ‘Vulnerable’ (VU), ‘Endangered’ (EN) and ‘Critically Endangered’ (CR) are regarded as threatened (Caetano et al., 2022). Additional species are evaluated and categorized as ‘Extinct in the Wild’ (EW), ‘Extinct’ (EX) or ‘Data Deficient’ (DD). The DD category is designated for species lacking adequate information to allocate them to any of the IUCN categories (Caetano et al., 2022).

However, a large portion of global biodiversity remains unassessed, that is, ‘Not‐Evaluated’ (NE) according to the red list. The existence of the NE category is the consequence of the lack of funds and other resources required by the resource‐intensive process of the red list evaluations, which rely heavily on voluntary contributions from experts and often entail multi‐participant and in‐person deliberations. As a result, DD and NE species do not receive priority in conservation decision‐making, despite the explicit instruction in red list guidelines that they should not be regarded as if they were not threatened (IUCN, 2019).

Due to the data requirements and the standardized process, red listing takes up a great deal of time and that is why only a fragment of the world's biological diversity has been assessed with coverage that varies across taxa. For example, 68% of well‐known vertebrates have been assessed at least once, as opposed to only 10% of plants (Holz et al., 2022), 2% of invertebrates, protists and fungi (Zizka et al., 2021). In addition, the proportion of assessed species is elevated in regions with available funding and experts (Bachman et al., 2019) and most of the current evaluations are outdated, that is, older than 10 years (Rondinini et al., 2014).

Currently, 7.7% (~23,000 species) of vascular plants worldwide are categorized as DD, along with 17.5% (~260,000 species) of the animal kingdom (IUCN, 2024) and 7.07% (1442 species) of the South African plants are also categorized as DD (South African National Biodiversity Institute [SANBI], 2024). With its 24,000 plant species, South Africa harbours ~10% of the world's plant richness (Rouget et al., 2004), of which more than 50% are endemic to the country. Worryingly, close to 10% of South African plants are threatened with the risk of extinction and these at‐risk plants are mostly found in the Fynbos biome, which is the most threatened biome on Earth (Cox & Underwood, 2011). In addition, South Africa's vegetation is grouped into nine terrestrial biomes, including the Nama Karoo Dessert Forest, Grassland, Albany thicket, Fynbos, Savannah, Indian Ocean Coastal Belt and Succulent Karoo (Rutherford et al., 2006). Also, of concern is the fact that 5% of vegetation types in South Africa are ‘Critically Endangered’, while 12% and 16% are ‘Endangered’ and ‘Vulnerable’, respectively (Driver et al., 2005). More interesting is the fact that South Africa hosts on its own one of the six renowned global floral kingdoms which is the Cape Floral Kingdom. Although it is the smallest one, the Cape Floristic Kingdom is the richest and most threatened of all (Cox & Underwood, 2011; Rouget et al., 2004).

Because of insufficient data on taxonomy, geographic distribution, population status or threats, numerous DD species are not undergoing any risk assessment. This makes it difficult to have a comprehensive understanding of the magnitude of threats to biodiversity (Bland et al., 2012). For example, we know that 25% of mammals with sufficient data are at risk of extinction (Hilton‐Taylor et al., 2009). However, this proportion becomes 21% if none of the DD species were considered threatened (Bland, Collen, et al., 2015) and if all DD species were deemed threatened, this proportion would rise to 36%, suggesting that continued efforts must be deployed to reveal the true status of DD species (Bland, Collen, et al., 2015). Establishing the most likely conservation status of NE and DD species is therefore crucial for a better understanding of the global status of biodiversity which would inform the protection efforts of threatened species (Bland, Collen, et al., 2015). Assessing the risk level of these species would demand substantial resources due to the expenses associated with surveys and red‐list evaluations (Bland, Collen, et al., 2015; Bland, Orme, et al., 2015).

To accelerate the red‐listing procedure and to overcome biases, approaches have been elaborated in recent years to computerize the procedure. These computerization approaches, after integrating species morphology, physiology, geographic distribution, molecular data as well as human disturbance, allow thousands of NE and DD species to be processed in minutes and be allocated their likely conservation status (Zizka et al., 2021). An example of such computerized approaches is the neural network (NN) which is a highly flexible family of artificial intelligence (AI) models utilized to execute regressions or classification tasks (LeCun et al., 2015). NN can virtually approximate any task, thus producing one of the most popular and important structures for predictions (Goodfellow et al., 2016).

For example, Bland, Collen, et al. (2015) and Bland, Orme, et al. (2015) estimated that employing AI predictive models based on species traits and geographical data could offer the most cost‐effective method for estimating the proportion of DD or NE facing extinction risk. Zizka et al. (2021) emphasized that these AI models prioritize speed, reproducibility, scope and individual case assessments, enabling the processing of thousands of data deficient or not evaluated species using publicly available data within minutes. According to Dauby et al. (2017), they provide initial red list evaluations using accessible data in accordance with IUCN criteria or forecast the extinction risk of species based on species traits (Pelletier et al., 2018). While each of these automation strategies possesses notable drawbacks (Walker et al., 2020), Zizka et al. (2021) estimated that they serve as beneficial instruments for addressing data gaps in datasets containing NE and DD species.

In the present study, our aim was to conduct an automated assessment of the extinction risk of DD and NE plant species in South Africa. South Africa is one of the megadiverse countries in the world (Skowno et al., 2019). The country is not only home to one of the six floral kingdoms but three of the worldwide biological diversity hotspots are also found within its borders (Skowno et al., 2019). With the current approximation of the worldwide flora at 379,881 taxa (Lughadha et al., 2016; Paton et al., 2008), ~10% of the plant diversity of the world is represented within South Africa's boundaries (Rouget et al., 2004). Furthermore, the country has an unparalleled number of endemic vascular plants, approximately 13,265 taxa representing 65% of South African flora that are globally recognized. This makes South Africa, in terms of endemism and diversity, an indisputable custodian of a significant store of the flora of the world. Hence, conserving South Africa's flora is of global interest and is also a significant challenge at the same time (Raimondo et al., 2013).

Our objectives are: (i) to evaluate the performance of different neural network classification (nn‐class) algorithms and (ii) to predict the conservation status of Not Evaluated and Data Deficient species in the South African flora.

## 2 MATERIALS AND METHODS

### 2.1 Study area

**FIGURE 1:**
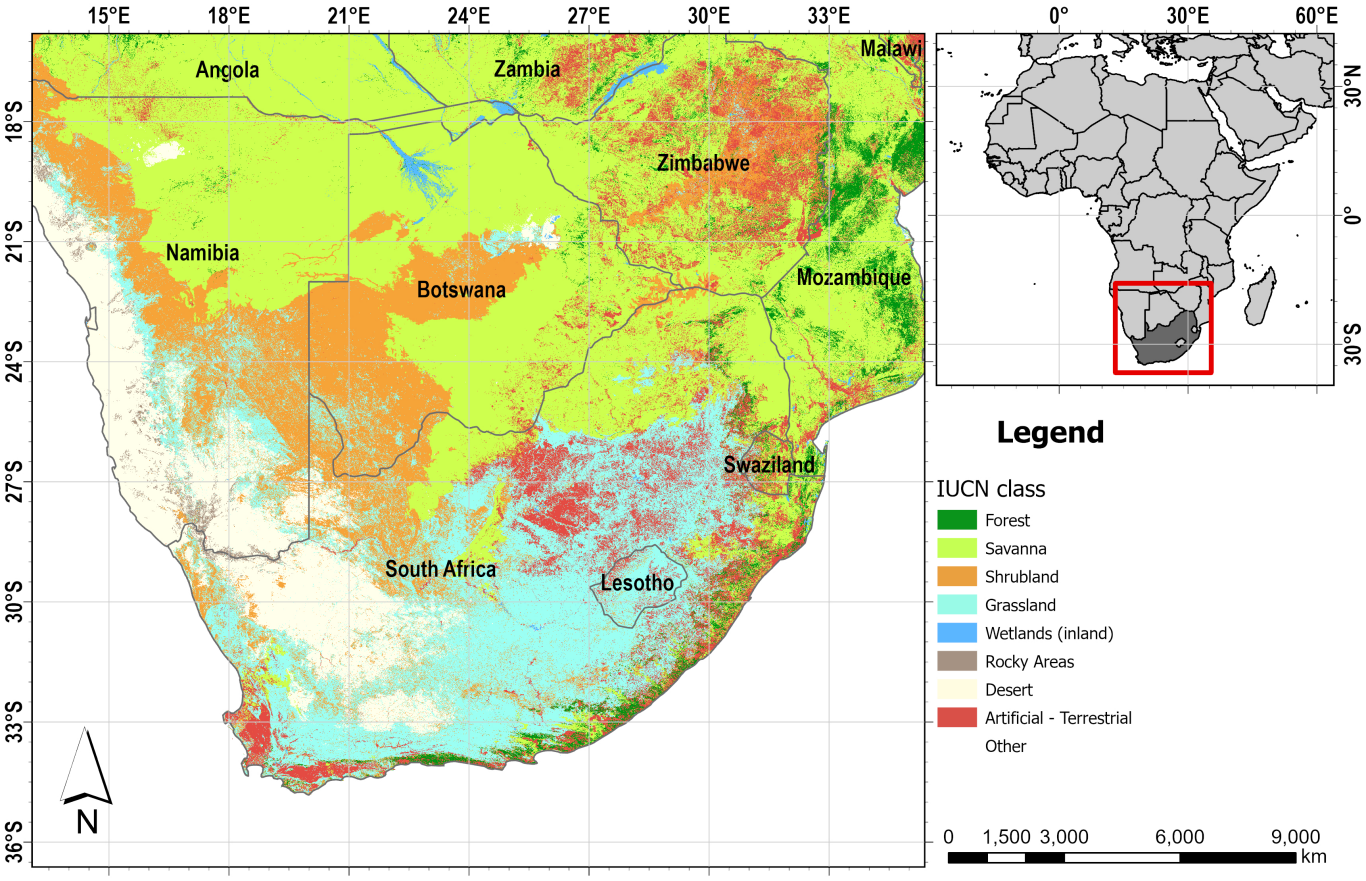
The location of South Africa in Africa shows the level 1 terrestrial habitat types according to the International Union for Conservation of Nature (IUCN) classification scheme (Jung et al., 2020).

South Africa is found at the southern extremity of Africa (Figure 1) with 3000 km of littoral. It is limited by the Indian Ocean in the east and the Atlantic Ocean in the west. Within a total land surface of 1,221,037 km^2^ latitudinally stretched from 22° S to 35° S and longitudinally from 17° E to 33° E, the country shares frontiers in the North with Namibia, Mozambique, Zimbabwe and Botswana, while Eswatini and Lesotho are two small independent countries landlocked within South Africa (Figure 1; Republic of South Africa, 2023).

Due to its oceanic surroundings, South Africa has a rather temperate climate, with significant variations across provinces. For example, the northern part of the Gauteng province has mild winters whereas the southern Gauteng experiences hot summers with intermittent rainstorms. The subtropical climate with high humidity is observed in the Kwazulu‐Natal province while the Eastern Cape province is characterized by the etesian climate with dry summers and rainy winters. Compared to a world rainfall average of 857 mm, the country's rainfall average is 500 mm. In South Africa, winter lasts from April to September and reaches temperatures of 0°C or less to 20°C at midday, whereas summer lasts from October to March and reaches temperatures of 15–35°C (South African Weather Service, 2024).

South Africa is famous for its exceptional biological diversity (Poulsen, 2020). The country's diversity and the uniqueness of its ecosystems and species make South Africa one of the 17 nations with the highest amount of biological diversity worldwide and among the top 3 when it comes to marine and plant species endemism (Skowno et al., 2019). The country's flora alone represents one‐tenth of the global botanical heritage, with some 24,000 species (Mamathaba et al., 2022). Most of the country's plant diversity is present in nine biomes (Mucina & Rutherford, 2006), including desert, Nama‐karoo, the Fynbos, Albany Thicket, Indian Ocean Coastal Belt, Forest, Grassland, Savana and Succulent Karoo (Finch & Meadows, 2019).

### 2.2 Data gathering

#### 2.2.1 Red list and species occurrence data

Data used for this study is comprised of species that are already RedListed (training data, Appendix S1), their occurrence records (Appendix S2) and the occurrence records of the species whose red listing categories need to be estimated (Appendix S3). We obtained the red list assessment of the South African vascular plants from the SANBI database (http://redlist.sanbi.org/, accessed in August 2023).

The classification of species in South Africa diverges somewhat from the IUCN red list categories by adding the categories ‘Rare’ and ‘Critically Rare’ and by dividing the data deficient category into data deficient insufficient information (DDD) and data deficient taxonomically uncertain (DDT) (Hoveka et al., 2020). The 2020 assessment included 20,381 species (South African National Biodiversity Institute, 2020): 14,020 least concern (LC) species, 547 not threatened (NT), 1559 vulnerable (VU), 886 endangered (EN), 404 critically endangered (CR), 414 DDD, 1028 DDT, 1246 rare, 166 critically rare, 75 critically endangered, possibly extinct (CR PE) and a total of 36 species that are extinct in the wild and extinct. To prepare a supervised learning model, we standardized the species obtained from the SANBI database into the IUCN's five most important threat categories: LC, NT, VU, EN, CR and therefore excluded species categorized as rare, critically rare, declining, extinct in the wild and those classified as DDT as well.

All the occurrence records data were retrieved from the Global Biodiversity Information Facility (GBIF, 2023) which contains 1,118,963 records for 21,692 species in South Africa, suggesting ~52 records per species (www.gbif.org, retrieved on 13.04.2023). Since records from public databank are fault prone (Zizka et al., 2020), we used the filtering options in excel to clean our data, retaining only records based on human observation and literature or living specimen.

Since 15 occurrence records are recommended as the minimum for automated assessment (Rivers et al., 2011), we kept species having at least 15 occurrences in the data. As a result, we obtained 404 LC species (2.8%), 155 NT species which represent 28% of the species assessed as NT by SANBI, 179 VU (11.4%), 150 EN (17%) and 68 CR (17%). Our data also includes 79 DD representing 19% of all species classified as DDD by SANBI and 5.49% of all DD species (DDD and DDT included). We also gathered 37 NE species from the Botanical Research and Herbarium Management System (BRAHMS) database. Overall, the total number of species that were analysed in the present study is 1072 (956 already assessed by SANBI used to train our model and 116 unassessed species used for predictions). Also, for our data, we recorded 112,066 occurrence points from GBIF, but after treatment (e.g. removal of species with fewer than 15 records, exclusion of naturalized individuals, hybrids, cultivars or breeds), it remained 96,938 records for the 1072 species analysed in this study.

### 2.3 Data analysis

R version 4.3.1 and Python were both used for our analyses. All the supplemental materials including R scripts used are available at https://github.com/samykandol/AI‐and‐conservation‐status‐in‐South‐African‐flora.git.

#### 2.3.1 Generating features and label preparation

We made use of the R package IUCNN (Zizka et al., 2021) to calculate features for each species based on species occurrence records (Appendix S4). The features extracted were mostly comprised of (i) geographic features such as mean latitude and longitude, extent of occurrences and area of occurrence, (ii) climatic (mean of 19 bioclimate features; Karger et al., 2017) and both features combined.

For label preparation, IUCNN converted the IUCN threat categories obtained from the SANBI red list into standardized numeric values. At the five class levels, LC was converted to 0; NT to 1; VU to 2; EN to 3 and CR to 4. However, at the binary level (2‐class prediction), not‐threatened species (LC and NT) were converted to 0 and threatened species (VU, EN and CR) to 1.

#### 2.3.2 Model training, testing and validation

The R package IUCNN offers a framework to access the Tensorflow Python library from within R (Abadi et al., 2015). With the IUCNN workflow, we trained the neural network classification (nn‐class) model using diverse types of features, including geographic information, climatic niche and a combination of both. The nn‐class employs a SoftMax activation function in its output layer, generating a vector of probabilities (Zizka et al., 2021)—a softMax activation function transforms raw outputs of the neural network into a vector of probabilities.

We classified species first into two distinct levels of projection (threatened [Thr] vs. not threatened [NThr]) and then into five red list categories (LC, NT, VU, EN and CR). During model training, we supervised validation loss to make sure the model was performing well. To prevent overfitting, we limited the number of epochs and adjusted the number of layers as needed.

Our training dataset was randomly separated into 2 (80% of entries for training and 20% for validation) and used a simple holdout method for cross‐validation. By setting a seed (in this case, the default: 1234), we ensured that the data is consistently divided into the same training, validation and test sets across different runs and model configurations (Chen et al., 2023; Zizka et al., 2021).

Following the initial tests, we configured the neural network (NN) architecture to explore different hyperparameter combinations. This allowed us to train six different nn‐class models with three types of hidden layers with 50 nodes, 40_20 nodes, 50_30_10 nodes, both without and with dropout regularization. For the estimation of prediction uncertainty and to prevent model overfitting, we used a dropout rate of 0.1 (Gal & Ghahramani, 2016).

#### 2.3.3 Prediction of extinction risk

We predicted the extinction risk at the 5‐class (detailed level: LC, NT, VU, EN and CR) and at the simplified binary level (not threatened vs. threatened). With the predicted features (Appendices S5–S8) extracted from the predicted occurrences (Appendix S3), we predicted the conservation status of 116 unassessed species in our dataset using the optimal trained models, selected based on the highest validation accuracy from a range of tested architectures. These models included diverse nn‐class model architectures with different numbers of hidden layers and subsets of features for both binary and detailed predictions.

**FIGURE 2:**
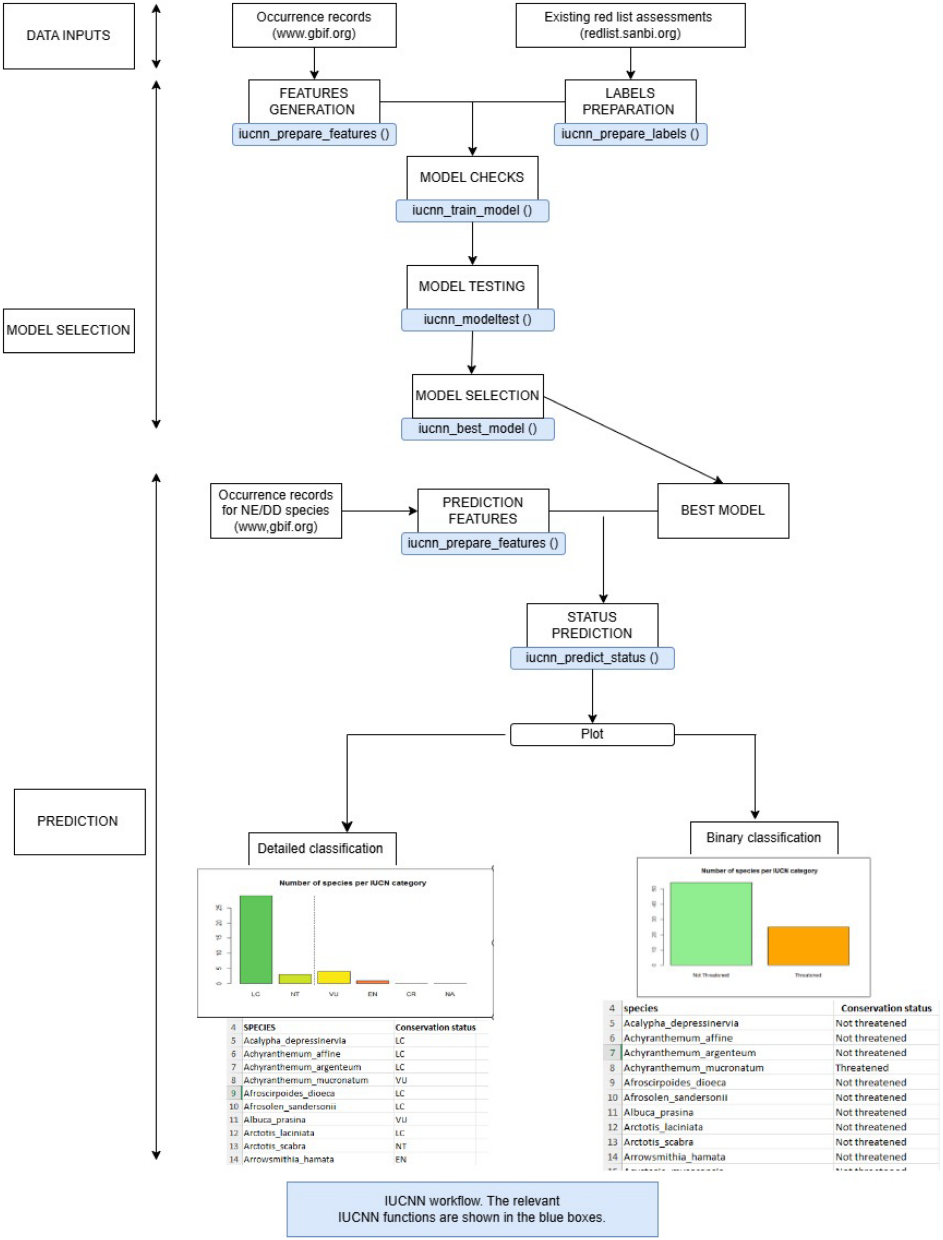
Summary of the methodology used in the present study from data collection and analysis.

Overall, the entire data collection and analysis are summarized in Figure 2.

#### 2.3.4 Geography of predicted threatened species

Here, two ancillary datasets were used. The former consisted of the Global Human Settlement Population Grid (GHS‐POP R2023A) by the Joint Research Centre (JRC) (Schiavina et al., 2023). The GHS‐POP R2023A was retrieved from the JRC's Visual analytics platform (https://ghsl.jrc.ec.europa.eu/ghs_pop2023.php, accessed 11/11/2023) as a spatial raster layer and contains information on the distribution of the residential population per 100 m cell (expressed as the number of people per cell). The latter was obtained from the South African Protected Areas Database (SAPAD, https://egis.environment.gov.za/protected_areas_register, accessed 11/11/2023), established by the National Environmental Management: Protected Areas Act (Act 57 of 2003).

The plugin, Biological Records Tool (https://www.fscbiodiversity.uk/qgisplugin/biorecstool, accessed 03/04/2024), was used to compute the species abundance and richness in QGIS 3.28. This tool presents a simple interface for users to aggregate biological records by grid. The species abundance and richness computations were aggregated at a 20 km × 20 km grid and Lambert Conformal Conic projection. The spatial distribution maps were created in ArcGIS Pro 3.2.

Using the occurrence data of species predicted by our model as threatened, we computed the proportion of species located outside and inside the protected areas. For this, the Pairwise Erase Tool in ArcGIS Pro 3.2 was used to intersect the point geometry of the predicted threatened DD Species and threatened NE species (i.e. input features dataset) with the Protected Areas dataset (i.e. erase features dataset).

## 3 RESULTS

### 3.1 Model selection and performance of the best model

We observed a decreasing training and validation loss (Figures S1 and S2), which indicates that the model is learning well from the data and on the data that it has not seen during training. Interestingly, there are no indications of overfitting in either case (detailed or binary classifications). This suggests that the model was able to generalize well to unseen data and was improving its predictive capability, as indicated by the performance on the validation dataset, even at a significant number of training epochs (172 and 125, respectively). The best training epochs for detailed classification and binary classification were 146 and 121, respectively (Figures S1 and S2).

As anticipated, binary assessments showed greater accuracy in classifying species into threatened and not‐threatened groups (with validation accuracy ranging from 79.08% to 83.6%), compared to the more detailed assessments (with validation accuracy ranging from 49.67% to 56.86%) (Table 1).

**TABLE 1:** Performance of the model with key parameters and all features included in the detailed and binary classifications.

Features	Model architecture	Detailed classification				Binary classification			
		Validation set		Test set		Validation set		Test set	
		Accuracy	Loss	Accuracy	Loss	Accuracy	Loss	Accuracy	Loss
Geographic	1 Layer	54.24	1.08546	46.07	1.25794	81.0458	0.43031	73.2984	0.54723
	2 Layers	52.28	1.0767	45.02	1.26628	80.3922	0.423	73.2984	0.55254
	3 Layers	52.94	1.0643	46.59	1.27024	81.0458	0.43113	74.3455	0.56434
	1 Layer + dropout	49.67	1.07435	45.02	1.28398	80.3922	0.42108	74.8691	0.56175
	2 Layers + dropout	53.59	1.06797	46.07	1.28935	80.3922	0.43042	74.3455	0.55825
	3 Layers + dropout	56.86	1.05917	46	1.30497	81.0458	0.42091	72.7749	0.56431
Climate	1 Layer	52.28	1.10914	45.54	1.346	79.085	0.46771	70.6806	0.58149
	2 Layers	50.98	1.10361	47.12	1.35109	82.3529	0.45687	69.6335	0.5903
	3 Layers	53.59	1.13375	46.07	1.33058	82.3529	0.44799	70.1571	0.59275
	1 Layer + dropout	50.32	1.09212	44.5	1.36187	79.7386	0.4445	68.5864	0.59285
	2 Layers + dropout	50.98	1.07808	42.93	1.37687	79.085	0.44595	69.1099	0.5892
	3 Layers + dropout	54.24	1.0994	44.5	1.35588	80.3922	0.44748	69.1099	0.62769
Geographic + climate	1 Layer	50.32	1.13511	46.59	1.2787	80.3922	0.45214	71.7277	0.54051
	2 Layers	53.82	1.082	44.5	1.25688	80.3922	0.46009	72.2513	0.52861
	3 Layers	55.86	1.0895	49.2	1.2705	81.0458	0.45277	76.4393	0.50609
	1 Layer + dropout	52.94	1.10551	45.02	1.27204	82.3529	0.4519	73.822	0.52011
	2 Layers + dropout	55.55	1.07742	45.02	1.29145	83.6601	0.430857	76.4397	0.51414
	3 Layers + dropout	52.28	1.0875	44.5	1.28766	81.0458	0.435179	73.822	0.55587

The nn‐class model (with 2 layers + dropout), which utilized both geographic + climate features for the two‐class prediction performed best, achieving a validation accuracy of 83.6% and a test accuracy of 76.4% (Table 1). Using this model, 91 out of 113 not‐threatened species in the test dataset were correctly classified as not‐threatened, resulting in an accuracy of 80.5%. Similarly, 55 out of 78 threatened species were accurately classified as threatened, resulting in an accuracy of 70.5% (Table 2).

**TABLE 2:** Confusion matrix of the best‐fitting model at the binary classification.

		Predictions		Input count (%)
		Not threatened	Threatened	
Test data	Not threatened	80.5	19.4	100
	Threatened	29.4	70.5	100

For the 5‐class prediction, the best fitting model was the nn‐class model with 3 layers + dropout, which used geographic features alone and achieved a cross‐validation accuracy of 56.86% and a test accuracy of 46% (Table 1). The least concern (LC) species were accurately identified in 82.6% of the cases and the accuracy was lower for the other classes (Table 3). In the majority of instances, species were allocated to an adjacent class, suggesting that the model retained the ability to accurately detect signals for these species.

**TABLE 3:** Confusion matrix of the best model at the detailed classification.

	Classes	Predictions					Input count (%)
		LC	NT	VU	EN	CR	
True values	LC	82.6	8	6.6	1.3	1.3	100
	NT	31.5	7.8	31.5	28.9	0	100
	VU	15.3	11.5	38.4	34.6	0	100
	EN	20	5.7	37.1	34.2	2.8	100
	CR	11.7	5.8	58.8	23.5	0	100

### 3.2 Predicted conservation status of DD and NE species

**FIGURE 3:**
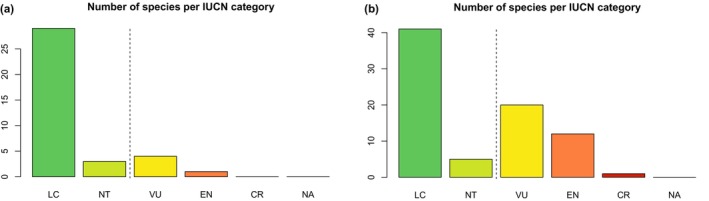
Predicted conservation status of vascular plants at 5‐class prediction with the NE species prediction (a) and DD species prediction (b).

**FIGURE 4:**
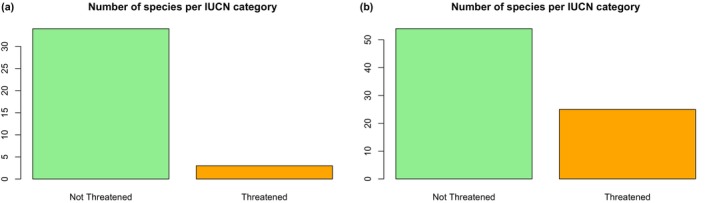
Conservation status of NE species with IUCNN at 2‐class prediction: NE species (a) and DD species (b).

We predicted the conservation status of 116 species (DD + NE) in our dataset with the best‐fitting model among the six model algorithms tested. At the detailed classification level, we employed the best‐performed model, which was the nn‐class incorporating geographic features alone (with 3 layers + dropout), to predict the conservation status of species categorized as not evaluated (NE) and data deficient (DD) separately. Figures 3 and 4 illustrate the conservation status of NE and DD species at the 5‐class prediction, while the comprehensive classification for each species can be found in Appendices S9–S11.

For the NE species, our model predicted that 29 species (78.37%) were classified as LC, 3 species (8.1%) as NT, 4 (10.8%) as VU and 1 (2.7%) as EN (Figure 3a). Meanwhile, the prediction for the DD species also predominantly fell into the LC category with 41 species (51.8%), whereas 5 species (6.3%) were classified as NT, 20 species (25.3%) as VU, 12 (15.1%) as EN and 1 species (1.2%) as CR (Figure 3b).

At the binary level, among the 37 NE species assessed, 34 species (91.8%) were predicted as not threatened while three species (8.1%) are currently threatened with extinction (Figure 4a). In the assessment of 79 species classified as DD, we found that 54 species (64.3%) are not threatened, whereas 25 species (31.6%) are deemed threatened (Figure 4b). These findings are detailed in Appendices S12–S14.

### 3.3 Spatial distribution of predicted threatened species

**FIGURE 5:**
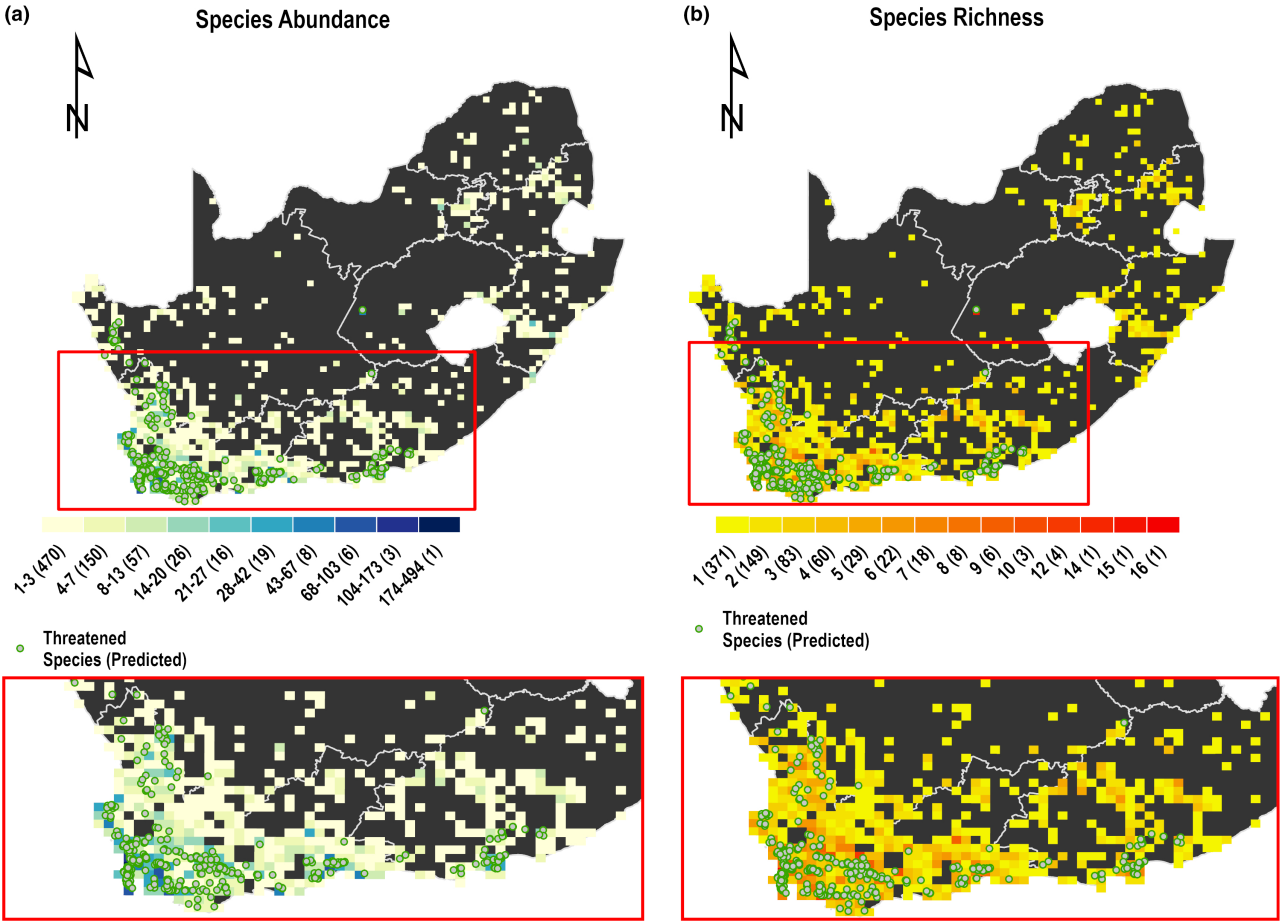
DD species abundance (a) and richness (b) overlaid with occurrence points of DD species predicted to be threatened (green dots). Abundance and richness are reported within 20 km grid cells. The frequency of occurrence of the respective grid is in brackets.

**FIGURE 6:**
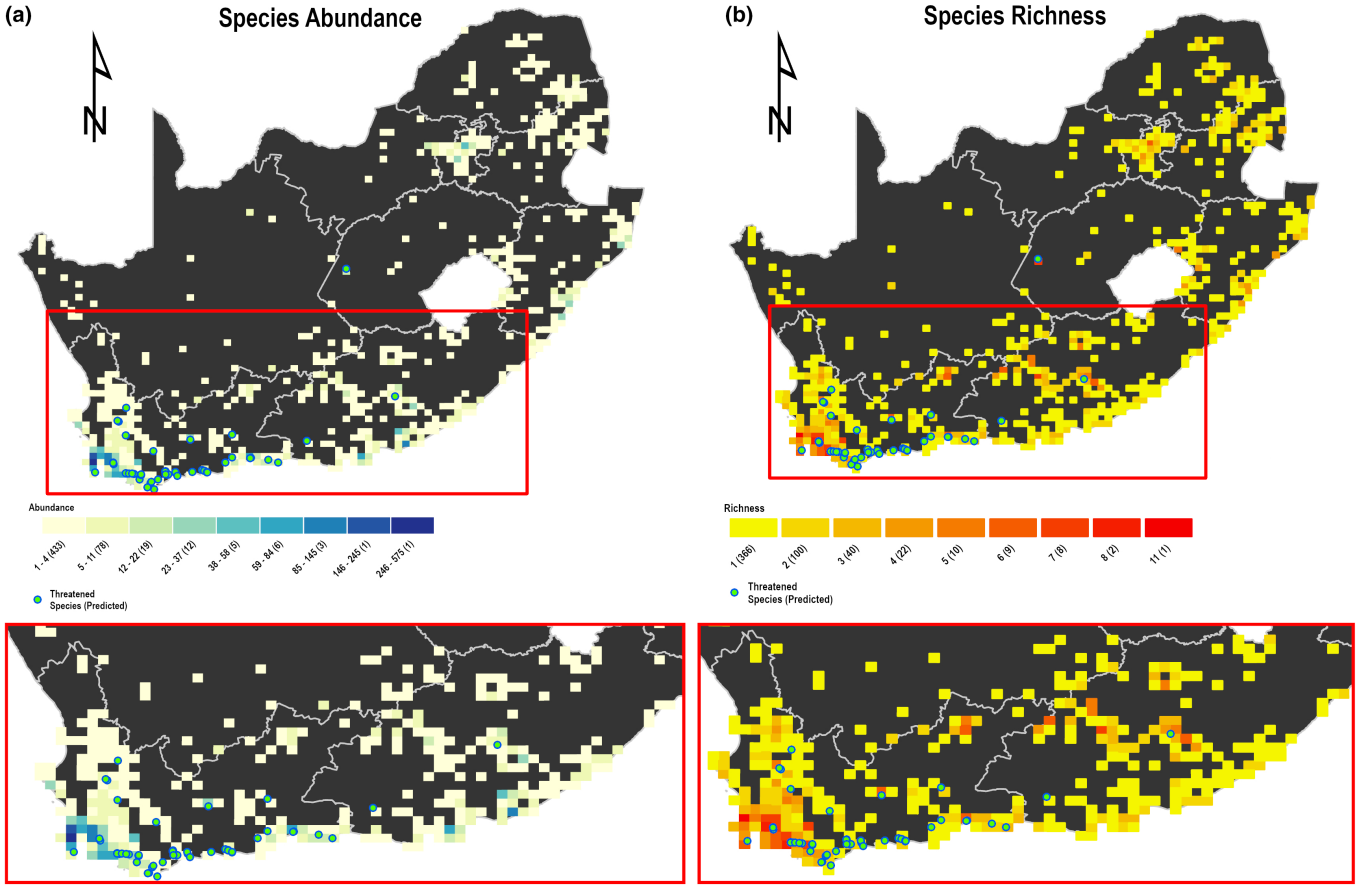
NE species abundance (a) and richness (b) overlaid with occurrence points of NE species predicted to be threatened. Abundance and richness are reported within 20 km grid cells. The frequency of occurrence of the respective grid is in brackets.

The spatial distribution maps of DD and NE species predicted to be threatened are presented in Figures 5 and 6, indicating that most DD (Figure 5) and NE species (Figure 6) predicted to be threatened are found in the southern part of the country in the Cape Floristic Region.

**FIGURE 7:**
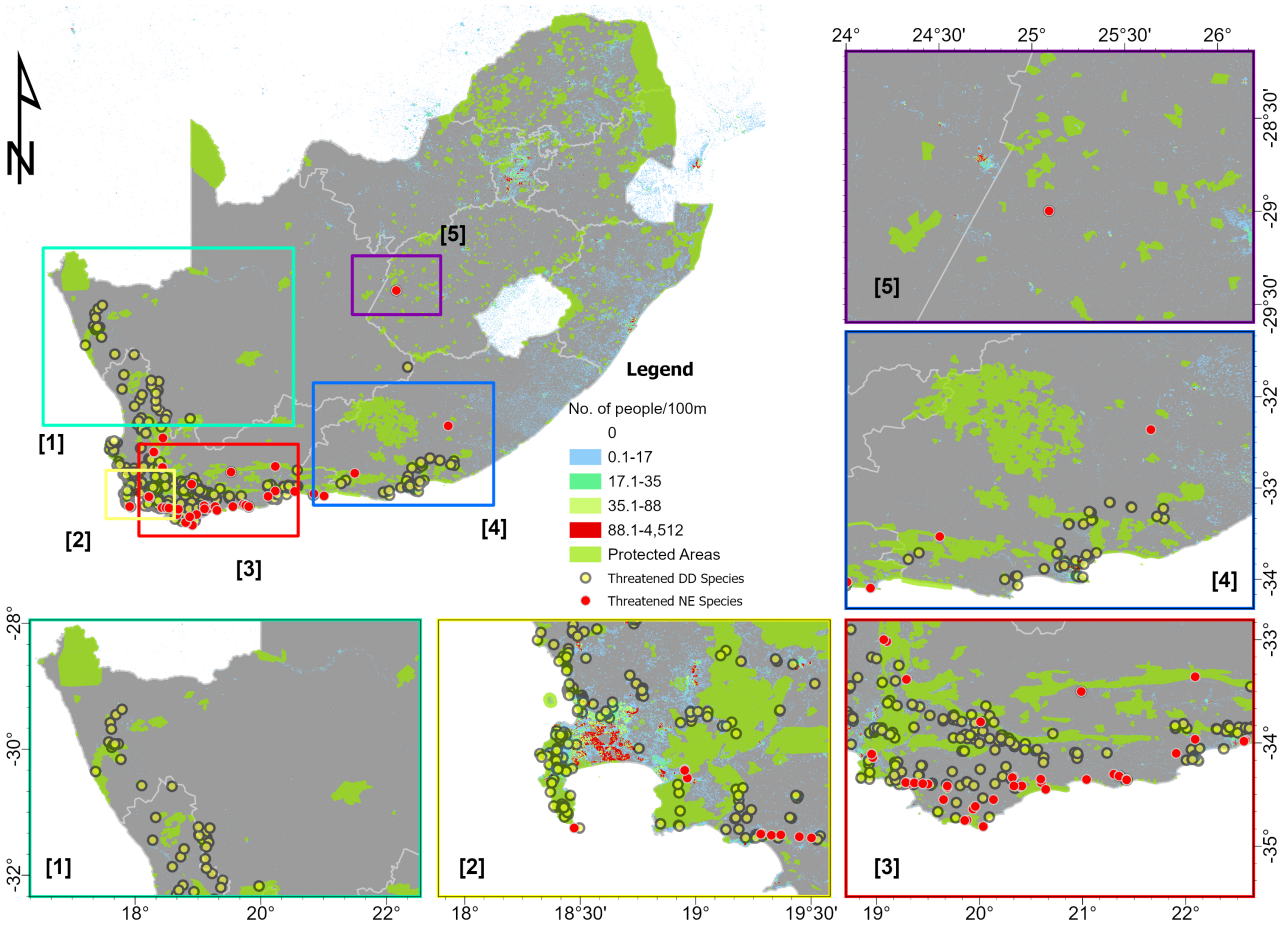
Threatened NE and DD species located inside and outside the nature reserves.

Interestingly, we found that all DD and NE species predicted to be threatened are found both inside and outside protected areas, but they are more abundant outside (73.33% of threatened DD species abundance) and a similar pattern is found for NE species abundance (79.07% of threatened NE species abundance is outside protected areas). These species predicted to be threatened are found in highly populated areas by humans (Figure 7).

## 4 DISCUSSION

A better understanding of species extinction threats assists in directing conservation efforts and preventing geographically and taxonomically biased judgements (Silva et al., 2022). In this study, we included an automated deep‐learning analysis to predict the conservation status of currently unassessed species in South Africa. To this end, we used a predictive approach (IUCNN) (Zizka et al., 2021). We trained the neural network classification (nn‐class) model on 956 species already assessed and different subsets of features with different model algorithms. We used nine geographic features following several authors (Chen et al., 2023; Silva et al., 2022; Stévart et al., 2019; Zizka et al., 2020), but also the mean of 19 bioclimatic variables (climate features; Chen et al., 2023; Silva et al., 2022; Zizka et al., 2020). These features, extracted exclusively from publicly available occurrence records, were used separately and in combination (Zizka et al., 2020).

Although species occurrences from open databases are prone to errors, Zizka et al. (2020) and Walker et al. (2020) demonstrated that adopting stricter filters to choose occurrences for species (by only taking into account recently recorded occurrences for features generation for example) did not significantly improve the prediction accuracy, it did significantly reduce the number of species that can be predicted. As a result, we decided to restrict record cleaning to simple automatic filters.

### 4.1 Reliability of our deep learning evaluation

The robust performance of deep learning models, indicated by low validation loss or higher prediction accuracy, ensures their capability to bridge the gap between research and real‐world applications. This reliability is crucial for converting theoretical advancements into practical solutions across different fields (Cazalis et al., 2022). We trained the nn‐class model using both the five IUCN classes (LC, NT, VU, EN and CR) and binary classification (Not threatened [NTh] vs. Threatened [Th]). Different configurations of hidden layers and a subset of features (climate, geographic and combined) were employed to predict the conservation status of vascular plants in SA.

At the detailed level (5‐class), the best performing model was the classifier that utilized geographic features alone with three layers + dropout, achieving a validation accuracy of ~57%. Our validation accuracy is similar (60%, 5‐class) to that reported for other plant groups assessed using similar techniques (orchids; Zizka et al., 2020) including trees (~67%; Silva et al., 2022). Furthermore, the test accuracy of our model (46%) for the 5‐class predictions follows a similar pattern: fish (50.7%; Chen et al., 2023) and orchids (64%; Zizka et al., 2020). However, our validation accuracy was notably lower than that reported for an animal group using the same technique (freshwater fish, 95.4%) in a recent study (Chen et al., 2023), suggesting that prediction of extinction risk may be taxonomy dependent (plants vs. animals).

The unbalanced representation of classes in the training set is one of the major issues with employing supervised learning techniques like neural networks. This is a natural result of the fact that some classes, like LC, have higher representation in IUCN RL data than others (Silva et al., 2022). To address this, we divided the five IUCN classes (detailed level) into two more general categories (Binary level: NTh vs. Th) following other authors (Chen et al., 2023; Silva et al., 2022; Stévart et al., 2019; Zizka et al., 2020).

At the binary level, our model performs better. Specifically, our best‐fitting model achieved a validation accuracy of 83.6% and a test accuracy of 76.43% using geographic features + climate features combined with the model algorithm of two layers + dropout, surpassing the accuracies reported by Zizka et al. (2020) using geographic features alone with three layers (81%), but lower than that reported in Silva et al. (2022) using biomes features (83.7%) and that of Chen et al. (2023) (99%) with a model algorithm comprised of one layer + Dropout and features such as biological traits, phylogenetic status, geographic sampling bias, human footprint, eco‐environment.

The differences in model performances might arise from differences in the algorithmic structures of the model (nn‐class) and the type of features used. For example, in the 5‐class predictions, Zizka et al.'s (2020) best model incorporated geographic + human footprint features, Silva et al. (2022) utilized biomes features whereas our study employed geographic features alone. For the 2‐class predictions, Zizka et al.'s (2020) best‐performing model used geographic features alone while ours used geographic + climate features. In contrast, Chen et al. (2023) enhanced the model's performance by incorporating additional feature datasets, such as biological traits and phylogenetic features. In the context of the binary classification, phylogenetic features and biological traits exert a substantial influence on model predictions. The incorporation of these features notably enhanced the model's performance, resulting in an increase in validation accuracy. However, the inclusion of these features did not influence the optimal model performance at the detailed classification (Chen et al., 2023).

### 4.2 Extinction risk of South African unassessed vascular plants

Based on the optimal trained models, we conducted separate predictions for the conservation status of 116 unassessed species (NE & DD) within our dataset, at both the detailed classification (5‐class) and binary classification (2‐class) levels. At the detailed level, the collective outcomes revealed the predominance of the LC category in our predictions (60.3%, 70/116 of classified species). This is not a surprise since similar patterns are reported in multiple studies: 75.7% for orchids (Zizka et al., 2020), 59% for freshwater fish (Chen et al., 2023), 59% for trees (Silva et al., 2022). The predominance of predicted LC among DD and NE species is likely because these species are abundant in remote areas that are difficult to access (Bland et al., 2017; Butchart & Bird, 2010) and therefore subject to reduced human pressure and thus have a lower risk of extinction (Zizka et al., 2021).

At the binary level, our model predicts that 32% (25) of DD species are threatened. Most studies that investigated, using similar methods (i.e. machine learning), the likely conservation status of DD species did so on animals. These studies predicted exceptionally high proportions of threatened species among DD animal species. For example, 64%–69% of DD mammals are predicted to be threatened (Bland, Collen, et al., 2015; Bland, Orme, et al., 2015; Jetz & Freckleton, 2015). This proportion is 50%–85% for amphibians (Borgelt et al., 2022) and for reptiles (Caetano et al., 2022). These findings suggest that a huge proportion of DD species may go extinct unnoticed if nothing is done (Bland, Collen, et al., 2015; Bland, Orme, et al., 2015). We need therefore more studies on unassessed plants to inform conservation actions, given that ~45% of global plant diversity is at risk of extinction (Bachman et al., 2024; Pelletier et al., 2018).

Our study also projects that 8% of NE species are threatened and this increases the overall proportion of threatened species in South Africa. Again, studies that investigated the likely conservation status of NE plant species are scant, while most focus on animal groups. For example, 22% of NE reptiles are predicted to be threatened (Bland & Böhm, 2016; Caetano et al., 2022). The few plant records targeted some lineages, predicting that 35% of NE bulbous monocots are threatened with risk of extinction (Darrah et al., 2017). When we combined all species lacking conservation status (DD + NE), our prediction indicates that 28 out of 116 species (24.1%) are threatened, implying ~76% of these species are not threatened. This trend (more non‐threatened than threatened) seems to be generalizable: 55% of all angiosperms are predicted to be non‐threatened (Bachman et al., 2024), ~67% of orchids are not threatened (Zizka et al., 2020) whereas the proportions of non‐threatened species are ~51% for trees (Silva et al., 2022) and ~72% for Chinese freshwater fish (Chen et al., 2023). Nevertheless, our model prediction that 24.1% of unassessed species are currently threatened with extinction risk is slightly higher compared to the worldwide average for plants based on the IUCN sampled red list index (20%) (Darrah et al., 2017) and higher compared to the SANBI's (2020) findings, which estimated that 16% of South Africa plants are currently threatened with extinction.

Since we were only able to assess species with at least 15 occurrence points, there is a possibility that an even greater proportion of species that were not assessed are at risk of extinction. That 24% of currently unassessed plants are threatened calls for action to protect approximately 3 out of 10 unassessed plants that are likely sliding into extinction. The question then becomes: Are unassessed species predicted to be threatened protected in the current network of South Africa's protected areas?

### 4.3 Are species predicted to be threatened covered in the existing network of protected areas?

Determining how biodiversity is spatially distributed across landscapes is a prerequisite for effective conservation measures (Margules & Presley, 2000). In megadiverse countries, the effectiveness of protected area networks in protecting biodiversity is rarely assessed (Paknia et al., 2015). When assessed, evidence indicates that these networks fail to adequately capture threatened taxa (Hoveka et al., 2020; Simaika & Samways, 2009; Tolley et al., 2019). Interestingly, we found that all DD and NE species predicted to be threatened are located inside protected areas, although they are more abundant outside protected areas too. This reveals the effectiveness of South Africa's protected areas in contributing to significantly protecting biodiversity. Even a recent study reported that the sampling effort of biodiversity is greater inside protected areas than outside (Hoveka et al., 2020), adding to the effectiveness of South Africa's protected areas in biodiversity knowledge generation (see also Tolley et al., 2019 for reptiles; Drinkrow & Cherry, 1995 for anurans; Evans et al., 2006 for birds).

However, since we found more abundant unassessed species that are potentially threatened outside protected areas, there is a need to (i) target species outside protected areas in IUCN risk assessment, (ii) increase the network of protected areas to cover potentially threatened species and (iii) as recently recommended, there is a need to increase sampling efforts of endemic plant diversity outside protected areas (Hoveka et al., 2020). Such efforts currently target animals, for example, endemic dragonflies (Simaika & Samways, 2009) and small mammals (Gelderblom & Bronner, 1995), indicating once more the disparity in biodiversity studies between animals and plants. However, the effectiveness of South Africa's protected areas in covering unassessed plants that are likely threatened should not hide some of their limitations. For example, Hoveka et al. (2020) identified 163 threatened endemic plants distributed outside any form of protected areas, particularly in the Fynbos in the Cape Floristic Region. It is also in this same region where all our unassessed plants predicted to be threatened are also located.

South Africa harbours almost 10% of the world's plant diversity and is the only country to host three biodiversity hotspots (Myers et al., 2000). This makes South Africa a key focus for world biodiversity conservation. However, its biodiversity is threatened with the risk of extinction, making risk assessment a priority. Given the complexity of risk assessment, several predictive approaches were proposed. We used a predictive approach (IUCNN) that consists of three fully connected neural networks and a CNN model. We trained our model on 956 species, based on different model algorithms and different subsets of features. The model was trained on nine geographic features (see also Chen et al., 2023; Silva et al., 2022; Stévart et al., 2019; Zizka et al., 2020), 12 climate features (Chen et al., 2023; Zizka et al., 2020) and on both features (see also Zizka et al., 2020). The model was used to predict the conservation status of 116 unassessed species. We found that 32% of DD and 8% of NE species are likely threatened and these species are also in protected areas, revealing the effectiveness of South Africa's network of protected areas. However, this effectiveness should not hide some of the limitations of South Africa's protected areas, which require a revision of the network to include threatened but unprotected species (Hoveka et al., 2020).

## AUTHOR CONTRIBUTIONS

**Bahati Samuel Kandolo:** Data curation (lead); formal analysis (lead); investigation (lead); methodology (lead); software (lead); visualization (lead); writing – original draft (lead). **Kowiyou Yessoufou:** Conceptualization (lead); funding acquisition (lead); investigation (equal); project administration (equal); supervision (lead); validation (lead); visualization (lead); writing – review and editing (lead). **Mahlatse Kganyago:** Methodology (supporting); resources (supporting); software (equal); visualization (supporting); writing – review and editing (supporting).

## CONFLICT OF INTEREST STATEMENT

None to declare.

## Supporting information


Appendices S1–S14

Figure S1

Figure S2

Data S1

## Data Availability

The data that supports the findings of this study and the R code are freely downloadable from GitHub repository at https://github.com/samykandol/AI‐and‐conservation‐status‐in‐South‐African‐flora.git.

## References

[ece370229-bib-0001] Abadi, M. , Agarwal, A. , Barham, P. , Brevdo, E. , Chen, Z. , Citro, C. , Corrado, G. S. , Davis, A. , Dean, J. , Devin, M. , Ghemawat, S. , Goodfellow, I. , Harp, A. , Irving, G. , Isard, M. , Jozefowicz, R. , Jia, Y. , Kaiser, L. , Kudlur, M. , & Zheng, X. (2015). TensorFlow: Large‐scale machine learning on heterogeneous systems . Software available from tensorflow.org.

[ece370229-bib-0002] Bachman, S. , Field, R. , Reader, T. , Raimondo, D. , Donaldson, J. , Schatz, G. E. , & Lughadha, E. N. (2019). Progress, challenges, and opportunities for red listing. Biological Conservation, 234, 45–55. 10.1016/j.biocon.2019.03.002

[ece370229-bib-0003] Bachman, S. P. , Brown, M. J. M. , Leão, T. C. C. , Lughadha, E. N. , & Walker, B. E. (2024). Extinction risk predictions for the world's flowering plants to support their conservation. New Phytologist, 2024(242), 797–808. 10.1111/nph.19592 38437880

[ece370229-bib-0004] Balding, M. , & Williams, K. J. (2016). Plant blindness and the implications for plant conservation. Conservation Biology, 30, 1192–1199. 27109445 10.1111/cobi.12738

[ece370229-bib-0081] Bland, L. M. , Bielby, J. , Kearney, S. , Orme, C. D. L. , Watson, J. E. M. , & Collen, B. (2017). Toward reassessing data‐deficient species. Conservation Biology, 31(3), 531–539. 27696559 10.1111/cobi.12850

[ece370229-bib-0007] Bland, L. M. , & Böhm, M. (2016). Overcoming data deficiency in reptiles. Biological Conservation, 204, 16–22.

[ece370229-bib-0008] Bland, L. M. , Collen, B. , Orme, C. D. L. , & Bielby, J. (2012). Data uncertainty and the selectivity of extinction risk in freshwater invertebrates. Diversity and Distributions, 18, 1211–1220.

[ece370229-bib-0009] Bland, L. M. , Collen, B. E. N. , Orme, C. D. L. , & Bielby, J. O. N. (2015). Predicting the conservation status of data‐deficient species. Conservation Biology, 29, 250–259. 10.1111/cobi.12372 25124400

[ece370229-bib-0010] Bland, L. M. , Orme, C. D. L. , Bielby, J. , Collen, B. , Nicholson, E. , & McCarthy, M. A. (2015). Cost‐effective assessment of extinction risk with limited information. Journal of Applied Ecology, 52, 861–870. 10.1111/1365-2664.12459

[ece370229-bib-0011] Borgelt, J. , Dorber, M. , Høiberg, M. A. , & Verones, F. (2022). More than half of data deficient species predicted to be threatened by extinction. Communications Biology, 5(1), 679. 35927327 10.1038/s42003-022-03638-9 PMC9352662

[ece370229-bib-0012] Brooks, T. , da Fonseca, G. A. B. , & Rodrigues, A. S. L. (2004). Species, data, and conservation planning. Conservation Biology, 18(6), 1682–1688.

[ece370229-bib-0082] Butchart, S. H. M. , & Bird, J. P. (2010). Data deficient birds on the IUCN red list: What don't we know and why does it matter? Biological Conservation, 143(1), 239–247.

[ece370229-bib-0013] Caetano, G. H. O. , Chapple, D. G. , Grenyer, R. , Raz, T. , Rosenblatt, J. , Tingley, R. , Böhm, M. , Meiri, S. , & Roll, U. (2022). Automated assessment reveals that the extinction risk of reptiles is widely underestimated across space and phylogeny. PLoS Biology, 20(5), e3001544. 10.1371/journal.pbio.3001544 35617356 PMC9135251

[ece370229-bib-0080] Cazalis, V. , Di Marco, M. , Butchart, S. H. M. , Akçakaya, H. R. , González‐Suárez, M. , Meyer, C. , Clausnitzer, V. , Böhm, M. , Zizka, A. , Cardoso, P. , Schipper, A. M. , Bachman, S. P. , Young, B. E. , Hoffmann, M. , Benítez‐López, A. , Lucas, P. M. , Pettorelli, N. , Patoine, G. , Pacifici, M. , … Santini, L. (2022). Bridging the research‐implementation gap in IUCN red list assessments. Trends in Ecology & Evolution, 37, 359–370. 35065822 10.1016/j.tree.2021.12.002

[ece370229-bib-0016] Chen, J. , Ding, C. , He, D. , Ding, L. , Ji, S. , Du, T. , Sun, J. , Huang, M. , & Tao, J. (2023). Assessing the conservation status of Chinese freshwater fish using deep learning. Reviews in Fish Biology and Fisheries, 2023(33), 1505–1521. 10.1007/s11160-023-09792-5

[ece370229-bib-0017] Cox, R. L. , & Underwood, E. C. (2011). The importance of conserving biodiversity outside of protected areas in Mediterranean ecosystems. PLoS One, 6, e14508. 21249126 10.1371/journal.pone.0014508 PMC3017544

[ece370229-bib-0018] Darrah, S. E. , Bland, L. M. , Bachman, S. P. , Clubbe, C. P. , & Trias‐Blasi, A. (2017). Using coarse‐scale species distribution data to predict extinction risk in plants. Diversity and Distributions, 23, 435–447.

[ece370229-bib-0076] Dauby, G. , Stévart, T. , Droissart, V. , Cosiaux, A. , Deblauwe, V. , SimoDroissart, M. , Sosef, M. S. M. , Lowry, P. P. , Schatz, G. E. , Gereau, R. E. , & Couvreur, T. L. P. (2017). ConR: An R package to assist large‐scale multispecies preliminary conservation assessments using distribution data. Ecology and Evolution, 7(24), 11292–11303. 10.1002/ece3.3704 29299301 PMC5743656

[ece370229-bib-0019] Drinkrow, D. R. , & Cherry, M. I. (1995). Anuran distribution, diversity and conservation in South Africa, Lesotho and Swaziland. African Zoology, 30(3), 82–90.

[ece370229-bib-0020] Driver, A. , Maze, K. , Rouget, M. , Lombard, A. T. , Nel, J. , Turpie, J. K. , Cowling, R. M. , Desmet, P. , Goodman, P. , Harris, J. , Jonas, Z. , Reyers, B. , Sink, K. , & Strauss, T. (2005). National spatial biodiversity assessment 2004: Priorities for biodiversity conservation in South Africa. SANBI.

[ece370229-bib-0021] Evans, K. L. , Rodrigues, A. S. , Chown, S. L. , & Gaston, K. J. (2006). Protected areas and regional avian species richness in South Africa. Biology Letters, 2(2), 184–188. 17148358 10.1098/rsbl.2005.0435 PMC1618914

[ece370229-bib-0022] Finch, J. M. , & Meadows, M. E. (2019). South African biomes and their changes over time. In J. Knight & C. M. Rogerson (Eds.), The geography of South Africa. World regional geography book series (pp. 57–69). Springer. 10.1007/978-3-319-94974-1_32

[ece370229-bib-0023] Forest, F. , Grenyer, R. , Rouget, M. , Davies, T. J. , Cowling, R. M. , Faith, D. P. , Balmford, A. , Manning, J. C. , Procheş, Ş. , van der Bank, M. , Reeves, G. , Hedderson, T. A. J. , & Savolainen, V. (2007). Preserving the evolutionary potential of floras in biodiversity hotspots. Nature, 445, 757–760. 17301791 10.1038/nature05587

[ece370229-bib-0024] Gal, Y. , & Ghahramani, Z. (2016). Dropout as a Bayesian approximation: Representing model uncertainty in deep learning. International Conference on Machine Learning.

[ece370229-bib-0025] GBIF.org . (2023). GBIF occurrence download . 10.15468/dl.bebu9p

[ece370229-bib-0026] Gelderblom, C. M. , & Bronner, G. N. (1995). Patterns of distribution and protection status of the endemic mammals in South Africa. African Zoology, 30(3), 127–135.

[ece370229-bib-0075] Goodfellow, I. , Bengio, Y. , & Courville, A. (2016). Deep learning. MIT Press.

[ece370229-bib-0028] Gordon, E. R. , Butt, N. , Rosner‐Katz, H. , Binley, A. D. , & Bennett, J. R. (2020). Relative costs of conserving threatened species across taxonomic groups. Conservation Biology, 34, 276–281. 31264731 10.1111/cobi.13382

[ece370229-bib-0030] Hilton‐Taylor, C. , Pollock, C. M. , Chanson, J. S. , Butchart, S. H. M. , Oldfield, T. E. E. , & Katariya, V. (2009). State of the world's species. In J.‐C. Vié , C. Hilton‐Taylor & S. N. Stuart (Eds.), Wildlife in a changing world. An analysis of the 2008 IUCN red list of threatened species (pp. 15–41). IUCN.

[ece370229-bib-0031] Holz, H. , Segar, J. , Valdez, J. , & Staude, I. R. (2022). Assessing extinction risk across the geographic ranges of plant species in Europe. Plants, People, Planet, 4, 303–311.

[ece370229-bib-0032] Hoveka, N. , van der Banka, M. , & Davies, T. J. (2020). Evaluating the performance of a protected area network in South Africa and its implications for megadiverse countries. Biological Conservation, 248, 108577.

[ece370229-bib-0033] International Union for Conservation of Nature (IUCN) . (2024). The IUCN red list of threatened species, version 2023‐1 . https://www.iucnredlist.org

[ece370229-bib-0034] IUCN Petitions Subcommittee . (2019). Guidelines for using the IUCN red list categories and criteria, version 14. IUCN.

[ece370229-bib-0036] Jetz, W. , & Freckleton, R. P. (2015). Towards a general framework for predicting threat status of data‐deficient species from phylogenetic, spatial and environmental information. Philosophical Transactions of the Royal Society of London. Series B, Biological Sciences, 370(1662), 20140016. 25561677 10.1098/rstb.2014.0016 PMC4290430

[ece370229-bib-0037] Jung, M. , Dahal, P. R. , Butchart, S. H. M. , Donald, P. F. , De Lamo, X. , Lesiv, M. , Kapos, V. , Rondinini, C. , & Visconti, P. (2020). A global map of terrestrial habitat types. Scientific Data, 7, 256. 10.1038/s41597-020-00599-8 32759943 PMC7406504

[ece370229-bib-0079] Karger, D. , Conrad, O. , Böhner, J. , Kawohl, T. , Kreft, H. , Soria‐Auza, R. W. , Zimmermann, N. E. , Linder, H. P. , & Kessler, M. (2017). Climatologies at high resolution for the earth's land surface areas. Scientific Data, 4, 170122. 28872642 10.1038/sdata.2017.122 PMC5584396

[ece370229-bib-0038] LeCun, Y. , Bengio, Y. , & Hinton, G. (2015). Deep learning. Nature, 521(7553), 436–444. 10.1038/nature14539 26017442

[ece370229-bib-0039] Lughadha, E. N. , Govaerts, R. , Belyaeva, I. , Black, N. , Lindon, H. , Allkin, R. , Magill, R. E. , & Nicolson, N. (2016). Counting counts: Revised estimates of numbers of accepted species of flowering plants, seed plants, vascular plants and land plants with a review of other recent estimates. Phytotaxa, 272(1), 82–88.

[ece370229-bib-0040] Mamathaba, M. P. , Yessoufou, K. , & Moteetee, A. (2022). What does it take to further our knowledge of plant diversity in the megadiverse South Africa? Diversity, 14, 748.

[ece370229-bib-0041] Margules, C. R. , & Presley, R. L. (2000). Systematic conservation planning. Nature, 405(67830), 243–253. 10821285 10.1038/35012251

[ece370229-bib-0044] Mucina, L. , & Rutherford, M. C. (Eds.). (2006). The vegetation of South Africa, Lesotho and Swaziland, Strelitzia 19. South African National Biodiversity Institute.

[ece370229-bib-0045] Myers, N. , Mittermeier, R. A. , Mittermeier, C. G. , Da Fonseca, G. A. , & Kent, J. (2000). Biodiversity hotspots for conservation priorities. Nature, 403(6772), 853–858. 10706275 10.1038/35002501

[ece370229-bib-0046] Paknia, O. , Sh, H. R. , & Koch, A. (2015). Lack of well‐maintained natural history collections and taxonomists in megadiverse developing countries hampers global biodiversity exploration. Organisms, Diversity and Evolution, 15(3), 619–629.

[ece370229-bib-0047] Paton, A. J. , Brummitt, N. , Govaerts, R. , Harman, K. , Hinchcliffe, S. , Allkin, B. , & Nic Lughadha, E. (2008). Towards target 1 of the global strategy for plant conservation: A working list of all known plant species—Progress and prospects. Taxon, 57, 602–611.

[ece370229-bib-0048] Pelletier, T. A. , Carstens, B. C. , Tank, D. C. , Sullivan, J. , & Espíndola, A. (2018). Predicting plant conservation priorities on a global scale. Proceedings of the National Academy of Sciences of the United States of America, 115(51), 13027–13032. 30509998 10.1073/pnas.1804098115 PMC6304935

[ece370229-bib-0051] Poulsen, Z. C. (2020). Megadiverse country: Introducing South Africa's biodiversity hotspots. Botanical Society of South Africa. BotSoc (botanicalsociety.org.za).

[ece370229-bib-0053] Raimondo, D. C. , Staden, L. V. , & Donaldson, J. S. (2013). Lessons from the conservation assessment of the South African megaflora. Annals of the Missouri Botanical Garden, 99, 221–230.

[ece370229-bib-0054] Republic of South Africa . (2023). Geography and climate . South African Government (www.gov.za).

[ece370229-bib-0055] Rivers, M. C. , Taylor, L. , Brummitt, N. A. , Meagher, T. R. , Roberts, D. L. , & Lughadha, E. N. (2011). How many herbarium specimens are needed to detect threatened species? Biological conservation, 144, 2541–2547. 10.1016/j.biocon.2011.07.014

[ece370229-bib-0057] Rondinini, C. , Marco, M. D. , Visconti, P. , Butchart, S. H. M. , & Boitani, L. (2014). Update or outdate: Long‐term viability of the IUCN red list. Conservation Letters, 7(2), 126–130. 10.1111/conl.12040

[ece370229-bib-0058] Rouget, M. , Reyers, B. , Jonas, Z. , Desmet, P. , Driver, A. , Maze, K. , Egoh, B. , & Cowling, R. M. (2004). National spatial biodiversity assessment 2004, technical report. Volume 1: Terrestrial component . South African National Biodiversity Institute.

[ece370229-bib-0059] Rutherford, M. C. , Mucina, L. , & Powrie, L. W. (2006). Biomes and bioregions of Southern Africa. Strelitzia, 19, 31–51.

[ece370229-bib-0060] Schiavina, M. , Freire, S. , Carioli, A. , & MacManus, K. (2023). GHS‐POP R2023A—GHS population grid multitemporal (1975–2030). European Commission, Joint Research Centre (JRC). http://data.europa.eu/89h/2ff68a52‐5b5b‐4a22‐8f40‐c41da8332cfe, 10.2905/2FF68A52-5B5B-4A22-8F40-C41DA8332CFE

[ece370229-bib-0061] Silva, S. V. , Andermann, T. , Zizka, A. , Kozlowski, G. , & Silvestro, D. (2022). Global estimation and mapping of the conservation status of tree species using artificial intelligence. Frontiers in Plant Science, 13, 839792. 10.3389/fpls.2022.839792 35574125 PMC9100559

[ece370229-bib-0062] Simaika, J. P. , & Samways, M. J. (2009). Reserve selection using red listed taxa in three global biodiversity hotspots: Dragonflies in South Africa. Biological Conservation, 142(3), 638–651.

[ece370229-bib-0063] Skowno, A. L. , Poole, C. J. , Raimondo, D. C. , Sink, K. J. , Van Deventer, H. , Van Niekerk, L. , Harris, L. R. , Smith Adao, L. , Tolley, K. A. , Zengeya, T. A. , Foden, W. B. , Midgley, G. F. , & Driver, A. (2019). National biodiversity assessment 2018: The status of South Africa's ecosystems and biodiversity . Synthesis report (pp. 1–214). South African National Biodiversity Institute, an entity of the Department of Environment, Forestry and Fisheries.

[ece370229-bib-0078] South African National Biodiversity Institute (SANBI) . (2020). Statistics: Red list of South African plants. Redlist.sanbi.org

[ece370229-bib-0065] South African National Biodiversity Institute (SANBI) . (2024). Statistics: Red list of South African plants . Redlist.sanbi.org

[ece370229-bib-0077] South African Weather Service . (2024). Annual state of the climate of South Africa 2023. Pretoria.

[ece370229-bib-0066] Stévart, T. , Dauby, G. , Lowry, P. P., 2nd , Blach‐Overgaard, A. , Droissart, V. , Harris, D. J. , Mackinder, B. A. , Schatz, G. E. , Sonké, B. , Sosef, M. S. M. , Svenning, J. C. , Wieringa, J. J. , & Couvreur, T. L. P. (2019). A third of the tropical African flora is potentially threatened with extinction. Science Advances, 5, eaax9444. 10.1126/sciadv.aax944 31799397 PMC6867875

[ece370229-bib-0067] Stuart, S. N. , Wilson, E. O. , McNeely, J. A. , Mittermeier, R. A. , & Rodríguez, J. P. (2010). Ecology: The barometer of life. Science, 328, 177. 20378803 10.1126/science.1188606

[ece370229-bib-0069] Tolley, K. A. , Weeber, J. , Maritz, B. , Verburgt, L. , Bates, M. F. , Conradie, W. , Hofmeyr, M. D. , Turner, A. A. , da Silva, J. M. , & Alexander, G. J. (2019). No safe haven: Protection levels show imperilled South African reptiles not sufficiently safe‐guarded despite low average extinction risk. Biological Conservation, 233, 61–72.

[ece370229-bib-0070] Vié, J. C. , Hilton‐Taylor, C. , & Stuart, S. N. (2009). Wildlife in a changing world—An analysis of the 2008 IUCN red list of threatened species. IUCN.

[ece370229-bib-0071] Walker, B. E. , Leão, T. C. C. , Bachman, S. P. , Bolam, F. C. , & Nic Lughadha, E. (2020). Caution needed when predicting species threat status for conservation prioritization on a global scale. Frontiers in Plant Science, 11, 520. 32411173 10.3389/fpls.2020.00520 PMC7199234

[ece370229-bib-0073] Zizka, A. , Andermann, T. , & Silvestro, D. (2021). IUCNN—Deep learning approaches to approximate species' extinction risk. Diversity and Distributions, 28, 227–241. 10.1111/ddi.13450

[ece370229-bib-0074] Zizka, A. , Silvestro, D. , Vitt, P. , & Knight, T. M. (2020). Automated conservation assessment of the orchid family with deep learning. Conservation Biology, 35(3), 897–908. 10.1111/cobi.13616 32841461

