# Supplementary material for: Effectiveness of South Africa's network of protected areas: Unassessed vascular plants predicted to be threatened using deep neural networks are all located in protected areas
Source: Ecol Evol. 2024 Sep 2;14(9):e70229. doi: 10.1002/ece3.70229 (PMC11368562; doi:10.1002/ece3.70229)
Supplement: Supplementary file 1 — Appendices S1–S14 [file ECE3-14-e70229-s004.zip › Legends.docx]

**Appendix S1**. Training labels

**Appendix S2**. Training occurrences

**Appendix S3**. Species for predictions and their occurrence records

**Appendix S4**. Geographic features

**Appendix S5**. Climate features

**Appendix S6**. Features for NE species predictions (Predict features NE)

**Appendix S7**. Features for DD species predictions (Predict features DD species)

**Appendix S8**. Predict features binary level for NE species

**Appendix S9**. Predict features binary level for DD species

**Appendices S10 and S11**. Conservation status of NE & DD species (Detailed classification)

**Appendices S12 and S13.** Conservation status of NE & DD species (Binary classification)

**Appendix S14**. All results
