# Supplementary figures and images for: Effectiveness of South Africa's network of protected areas: Unassessed vascular plants predicted to be threatened using deep neural networks are all located in protected areas
Source: Ecol Evol. 2024 Sep 2;14(9):e70229. doi: 10.1002/ece3.70229 (PMC11368562; doi:10.1002/ece3.70229)

## CV-fold 1

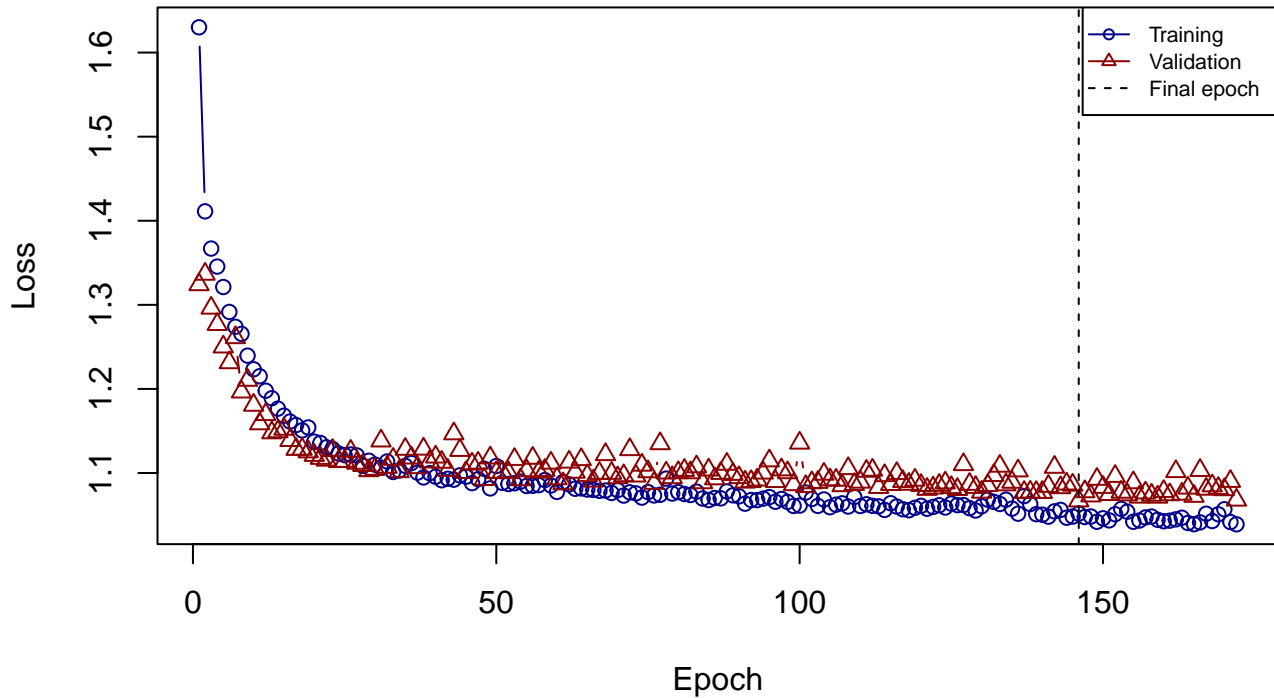

Supplement: Supplementary file 2 — Figure S1 [file ECE3-14-e70229-s001.pdf]

## CV-fold 1

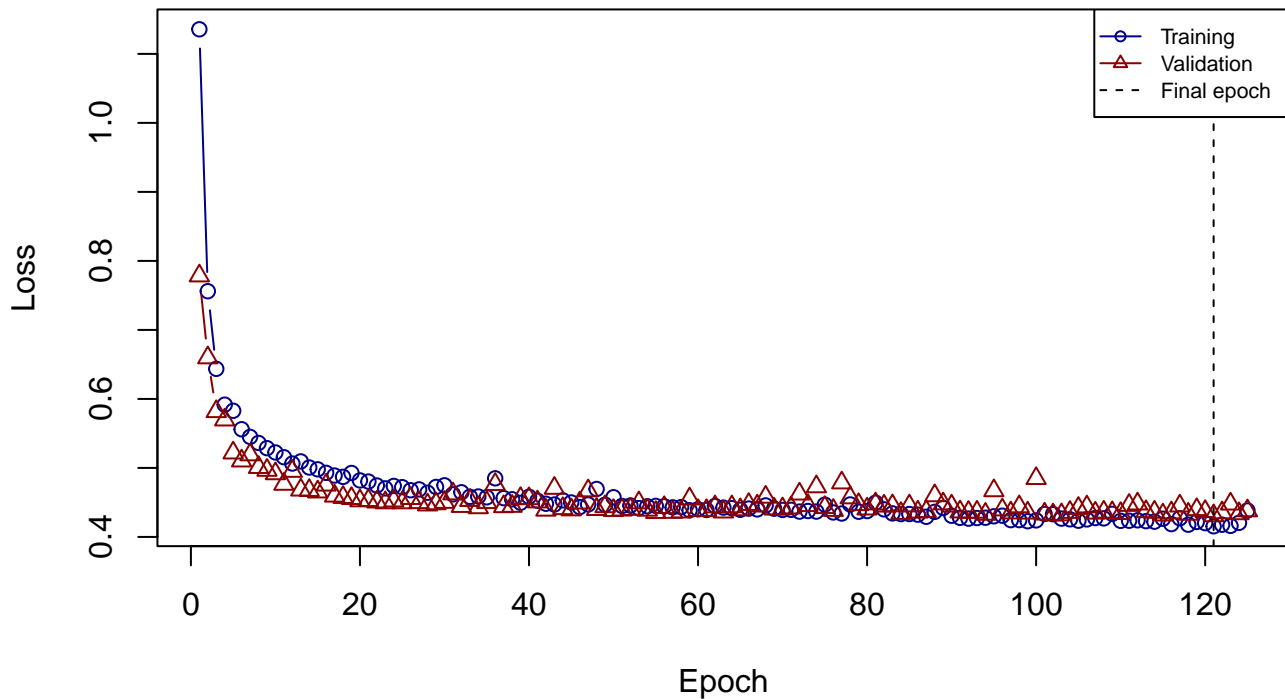

Supplement: Supplementary file 3 — Figure S2 [file ECE3-14-e70229-s002.pdf]
